# Transcriptome-Based miR156-Mediated Expression Dynamics of SPL Transcription Factors During Vegetative to Reproductive Transition in Spinach

**DOI:** 10.3390/plants14223543

**Published:** 2025-11-20

**Authors:** Ehsan Khalid, Yutong Zheng, Tengqi Wang, Lingmin Cai, Ray Ming

**Affiliations:** 1 Corp Genetics and Breeding, College of Agriculture, Fujian Agriculture and Forestry University, Fuzhou 350002, China; 2 Centre for Genomics and Biotechnology, Fujian Provincial Key Laboratory of Haixia Applied Plant Systems Biology, Fujian Agriculture and Forestry University, Fuzhou 350002, China

**Keywords:** phase transition, SPL, *SpmiR156*, spinach

## Abstract

Vegetative to reproductive phase transition is an important developmental process in flowering plants, regulated in part by microRNAs that repress target genes post-transcriptionally. However, the role of miR156 and its target Squamosa Promoter Binding Protein-Like (SPL) transcription factors remains poorly understood. In this study, we identified 14 SPL gene members in spinach and analyzed their expression during phase transition. Genome-wide identification and transcriptome-based analysis revealed that 11 of these genes are likely direct targets of *SpmiR156a/b/c/d*, with binding sites confirmed by sequence-based interaction prediction. Expression profiling showed that *SpSPL3* and *SpSPL8*, which are strongly repressed during vegetative growth, were significantly regulated during the transition phase. Gene Ontology (GO) and promoter cis-element analyses support that SPL genes are involved in hormonal pathways and floral development Quantitative Real-Time Polymerase Chain Reaction (qRT-PCR) further validated the transcriptome expression patterns of key SPL genes. Together, these findings outline a regulatory framework in which *SpmiR156* modulates *SpSPL* gene activity to control developmental phase change in spinach, highlighting both the expansion and functional diversification of the SPL gene and the central role of *SpmiR156* in vegetative to reproductive transition.

## 1. Introduction

The transition from a vegetative to reproductive phase is a crucial event in the life cycle of flowering plants, as it begins with vegetative growth, followed by the reproductive phase [1]. Vegetative growth is recognized as the juvenile growth of a plant while reproductive growth requires a phase shift period for the onset of the adult phase of flowering plants [2,3]. Transitions between developmental phases include changes in morphological and physiological traits, which are reflected in tissue size, shape, and identity [1,4,5]. For example, the vegetative to adult transition is characterized by the transformation of leaf abaxial trichomes, an increased leaf length/width ratio, and serration in Arabidopsis. Furthermore, vegetative to reproductive phase transition is characterized by the conversion of the vegetative Shoot Apical Meristem (SAM) to the Inflorescence Meristem (IM) [6,7].

MicroRNAs (miRNAs), a class of small endogenous non-coding RNAs involved in the developmental phase transition in plants by negatively regulating the expression of their target genes at post-transcriptional levels [8]. Various studies have shown that developmental phase transitions can be genetically regulated in part by miRNAs, which participate in complex genetic networks controlling plant development stages [9,10,11,12]. MiRNAs are usually around 18 to 24 nucleotides in length [13]. The first miRNA to be identified was lin4-RNA, which is a key regulator controlling developmental control in nematode (*Caenorhabditis elegans*) [9]. Since the discovery of plant miRNAs in Arabidopsis, a large number of miRNAs have been continuously identified in plants in recent years [14,15]. Plant genomes typically possess several hundred miRNA-targeted genes, many of which exist as families [16,17]. *MiR156* plays a distinguished role in phase transition by being highly expressed at the seedling stage and down-regulated with age, acting as a master negative regulator of phase transition genes in plants [1,11,12]. MicroRNA156 is a highly conserved small RNA in plants that regulates plant growth and development by targeting Squamosal Promoter Binding Protein-Like (SPL, also known as SBP-box) TFs [18].

SPLs are the transcription factors originally confirmed in *A. thaliana* to regulate developmental phase transitions, which are accompanied by targeting miR156 [19]. SPLs were first identified in *Antirrhinum majus* (snapdragon), encoding a plant-specific TF with a conserved SBP domain, by which SPL can recognize and bind specifically to the promoter region of target genes to regulate plant development [20,21,22]. The SPL family contains multiple members, such as 16 in *Arabidopsis thaliana* [23], 19 in *Oryza sativa* (rice) [24], and 56 in *Populus tomentosa* (poplar) [25]. Some of them were found to be involved in the transition of developmental phases mediated by miR156. In *Arabidopsis thaliana*, the overexpression of *miR156b* produced more leaves and delayed flowering, accompanied by the down-regulation of *SPL2/3/4/5/6/9/10/11/13/15* genes [26]. Contrarily, the decrease in the level of *miR156a/miR156* caused the up-regulation of several SPL genes, thereby producing fewer leaves and promoting juvenile to adult transition [19,27]. *MiR156a/c* mutation up-regulated the expression of *SPL2/3/9/10/11/13/15* genes, thus accelerating the production of leaf trichomes, resulting in promoting the juvenile to adult transition in Arabidopsis [6]. In rice, the *miR156-SPL14* module was found to be involved in the control of developmental phase transitions [28,29]. The overexpression of a *miR156*-resistant *OsSPL14* caused a decrease in tiller number, but an increase in plastochron and acceleration in floral transition [30]. In transgenic rice overexpressing *miR156f*, the decreased expression of *OsSPL14* together with *OsSPL3* and *OsSPL12* produced more tillers and displayed dwarfism [31]. Another report indicated that the overexpression of two *miR156* genes (*miR156b/h*) resulted in reduced panicle size and, in particular, delayed flowering. Contrary to the expression trend of the *miR156b/h*, *OsSPL2/12/13* and *OsSPL16/18* showed decreased mRNA levels in the flag leaves and panicles of transgenic plants, respectively, while *OsSPL14* was down-regulated in both flag leaves and panicles of transgenic rice [32]. In particular, the overexpression of *miR156* reduced the expression of *SPL3* and *SPL9*, which drastically prolonged the juvenile phase [33].

Despite the conserved role of the *miR156-SPL* module in regulating phase transitions across plants, its dynamics in dioecious species like spinach remain unexplored. Here, we integrate genome-wide identification, transcriptome-based *miR156*-mediated expression dynamics of SPL TFs profiling, and qRT-PCR validation to dissect this regulatory network across distinct developmental stages (vegetative, transition, and reproductive phases) in dioecious (XX ♀, XY ♂) spinach (*Spinacia oleracea* L., 2n = 12). We identified *SPL* genes retaining *miR156a/b/c/d* binding sites and demonstrate that *SpmiR156* down-regulation causes *SpSPL* repression and triggers floral transition. Our findings provide a molecular proposal for controlling phase transitions in spinach with implications for optimizing spinach flowering time.

## 2. Results

### 2.1. Classification of Spinach Plant Growth Stages

**Figure 1:**
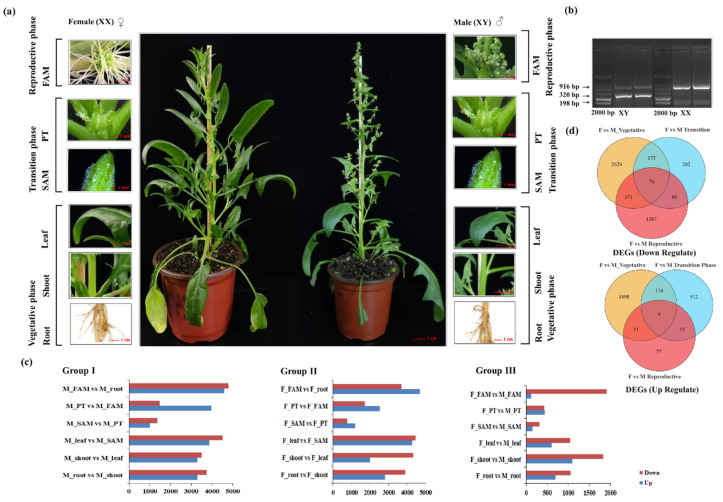
Morphological characteristics and expression profile of dioecious spinach at different developmental stages. (**a**) Vegetative, transition, and reproductive phase tissues, including root, shoot, leaves, SAM, PT, and FAM in dioecious spinach; (**b**) molecular identification of male and female plants using sex linked markers. PCR results T11A and SpoX amplify from XY and XX genomic templates. T11A amplifies a 916 bp band from an autosomal target in each sex type, but only the 320 bp male-specific band from XY. SpoX amplifies a 198 bp product from the XX template; (**c**) up-/down-regulating Differentially Expressed Gene (DEGs) analysis across the three developmental stages; (**d**) Venn diagram analysis illustrating Differentially Expressed Genes (DEGs) and overlapping sets of up-regulated and down-regulated genes across the vegetative, transition, and reproductive stages of spinach. SAM, shoot apical meristem; PT, phase transition meristem; FAM, flower apical meristem.

Spinach plants were systematically classified into three distinct developmental stages, vegetative, transition, and reproductive, to investigate the *miR156*-mediated expression dynamics of SPL TFs during developmental progression. This stage-wise classification was essential for elucidating the role of *SpmiR156*-*SpSPL* regulatory modules in vegetative to reproductive phase transition. Morphological characterization of dioecious spinach revealed clear stage-specific differences in both male and female plants, with root, shoot, and leaf tissues representing the vegetative stage, the Shoot Apical Meristem (SAM) and Phase Transition meristem (PT) tissues defining the transition stage, and the Floral Apical Meristem (FAM) representing the reproductive stage (Figure 1a). Molecular identification of sex types was conducted using sex linked markers, ensuring accurate differentiation between male and female plants prior to transcriptomic profiling (Figure 1b). Differentially Expressed Genes (DEGs) analysis across the three developmental stages revealed distinct transcriptomic shifts in both sexes (Figure 1c). Venn diagram analysis identified Differentially Expressed Genes (DEGs) and overlapping sets of up-regulated and down-regulated genes across the vegetative, transition, and reproductive stages in male and female plants. A total of 2629, 282, and 1387 down-regulated DEGs were identified at the vegetative, transition, and reproductive stages, respectively, with 76 shared across all stages. Up-regulated DEGs numbered 1898, 512, and 77 at the corresponding stages (Figure 1d). This developmental framework enabled the dissection of SPL gene expression patterns under the regulatory influence of miR156, providing key insights into the molecular mechanisms governing growth phase transitions in spinach.

### 2.2. Identification and Protein Physicochemical Properties of SPL Members in Spinach

To identify *SPL/SBP* genes in spinach, we analyzed the spinach reference genome (Sp75 genome; released 2021) (http://spinachbase.org/ftp/genome/Sp75/ accessed on 15 December 2024) [34] using the HMM profile of the SPL domain “PF03110” and performed BLASTp searches with SPL protein sequences from Arabidopsis (*Arabidopsis thaliana*) and oat (*Avena sativa*). Low-quality and redundant sequences lacking a start or stop codon were excluded to ensure high-quality gene models. Finally, 14 unique SPL proteins were identified and designated as *SpSPL*1 to *SpSPL*14, each containing a conserved SBP domain (Table S1). Analysis of their physiochemical properties revealed that the coding sequences (CDs) of *SpSPL* genes ranged from 330 to 3237 base pairs, encoding proteins between 109 and 1078 amino acids (Table 1). The predicted isoelectric points (PI) of these proteins ranged from 5.67 to 9.59, and their molecular mass varied from 12.61 to 126.61 kDa. Subcellular localization prediction (Table 1) indicated that 11 of the 14 *SpSPL* proteins were localized to the nucleus, two to the plasma membrane, and one to the endomembrane system. All *SpSPL* proteins exhibited instability indices greater than 40, ranging from 46.65 to 75.80, suggesting they are generally unstable and potentially prone to rapid degradation. *SpSPL* with the highest instability index of 75.80 was predicted to be the most unstable, while the one with the lowest index of 46.65 may possess marginal stability. The liphatic indices of the *SpSPL* proteins ranged from 42.02 to 81.31, with higher values indicating greater thermostability. Additionally, all *SpSPL* proteins had negative Grand Average of Hydropathy (GRAVY) scores, ranging from −0.353 to −1.093, indicating a hydrophilic nature. The most hydrophilic protein (GRAVY = −1.093) was likely to have strong solvent affinity, while the least hydrophilic (GRAVY = −0.353) may contain partial hydrophobic regions (Table 2).

### 2.3. Phylogenetic Relationship of SPL Gene Members

**Figure 2:**
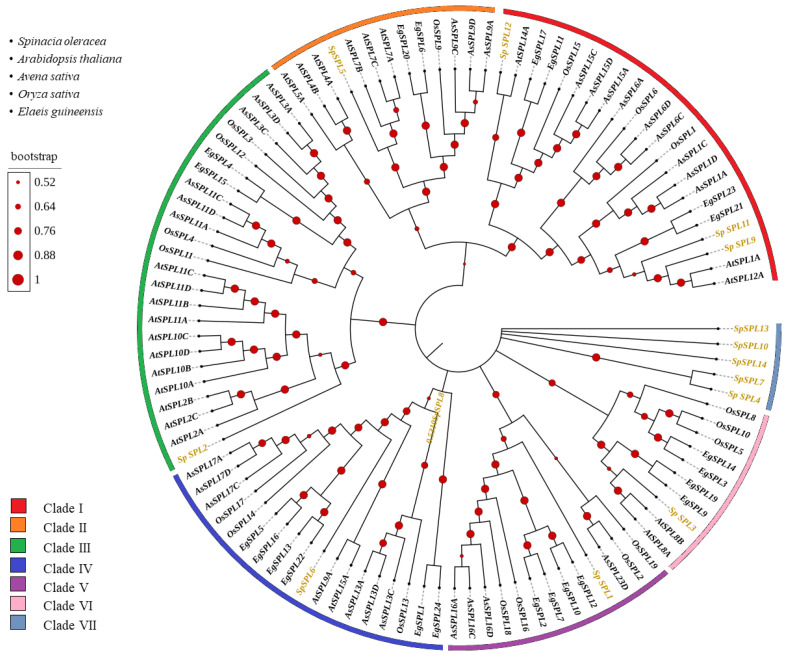
Phylogenetic analysis of *SpSPL* gene members from spinach. Phylogenetic tree of *SpSPL* gene members from spinach, Arabidopsis, rice, oat, and oil palm using the maximum likelihood (RaxML) method with 1000 bootstrap replicates on nodes marked by symbols and visualized in iTOL, categorized into I to VII clades with distinct colors. *Sp*: *Spinacia oleracea*; *At*: *Arabidopsis thaliana*; *As*: *Avena sativa*; *Os*: *Oryza sativa*; *Eg*: *Elaeis guineensis*.

The evolutionary relationships of SPL (Squamosa Promoter-Binding Protein-Like) gene members in spinach were analyzed using protein sequences containing the SPL domain from *Arabidopsis*, rice (*Oryza sativa)*, oat (*Avena sativa*), and oil palm (*Elaeis gineensis*) (Figure 2). Fourteen *SpSPL* members were classified into seven major clades based on topology and high-confidence bootstrap support values. In Clade I, three *SpSPL* members (*SpSPL9*, *SpSPL1*, and *SpSPL12*) were clustered with *Arabidopsis* (*AtSBP1A* and *AtSBP12A*) and oil palm (*EgSPL21* and *EgSPL23*) SPL members, supported by 0.9940 bootstrap values, indicating strong evolutionary conservation. Clade II included only one *SpSPL* member (*SpSPL*5), which was grouped with *AtSBP7A/b/c*, *EgSPL20/6*, *OsSPL9*, and *AsSPL9A/C/D*. In Clade III, *SpSPL*2 was grouped with *AtSBP2/10/11*, with a bootstrap value of 0.9960. Clade IV included *SpSPL*6 with *AtSBP9A/15*, *EgSPL22/13/16/15*, and *OsSPL14*. In Clade V, *SpSPL*1 was clustered with *EgSPL7/12/10/2*, *OsSPL16/18* and *AsSPL16A/C/D*. Clade VI included *SpSPL*3, which was a cluster with Arabidopsis *AtSBP8A/B*, and oil palm *EgSPL9/19*. Clade VII was a small subgroup of *SpSPL*4, *SpSPL*7, *SpSPL*10, *SpSPL*13, and *SpSPL*14 without any direct Arabidopsis, rice, oat, and oil palm.

### 2.4. Multiple Protein Sequence Alignment, Domain Confirmation, and Conserved Motif Analysis

**Figure 3:**
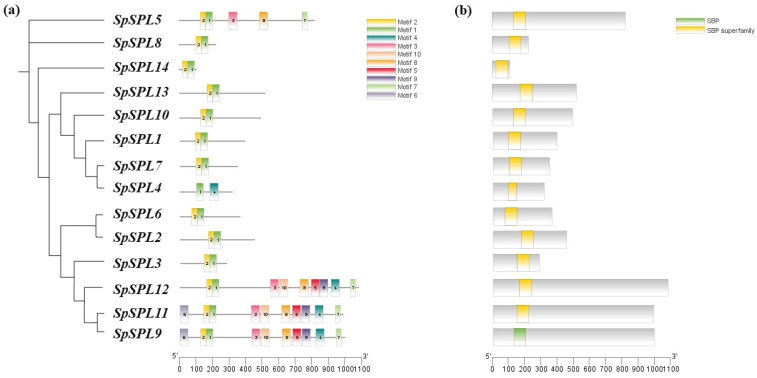
Gene structure, conserved motif distribution, and domain analysis of *SpSPL* gene members in spinach. (**a**) Distribution of conserved motifs identified using MEME. Different motifs (1–10) are represented in distinct colors, with gene names and group classifications displayed on the left. The ruler at the bottom indicates the amino acid sequence lengths; (**b**) conserved domain analysis of *SpSPL* gene members performed using the Conserved Domain Database (CDD) search tool, and domain architectures were compared across *SpSPL* members to confirm the presence of the SBP domain characteristic of this transcription factor family.

We conducted multiple protein sequence alignments, domain and conserved motif analyses, and gene structure examinations. Multiple sequence alignment of the *SpSPL* protein sequences confirmed the presence of the conserved SPL domain “PF03110” (Figure S1a,b). In conserved motifs analysis, a total of 10 conserved motifs were identified and named as motifs 1 to 10 (Figure 3a). The distribution of these motifs closely represents the phylogenetic classification of fourteen *SpSPL* members. Motifs 1 and 2 correspond to the highly conserved SBP DNA-binding domain, which is present in all *SpSPL* members. *SpSPL*1/2/3/6/7/8/10/13/14 contains only these two motifs (motifs 1 and 2). *SpSPL*9/11/12 contains the most complex motif architecture, comprising up to seven distinct motifs. The diversity in motif architecture suggests the functional specialization of *SpSPL* genes during spinach growth and development. Domain analysis using the CDD database confirmed the conserved SBP domain in all *SpSPL* proteins (Figure 3b). Most proteins harbored a single SBP domain, typically located near the Na-terminus, while *SpSPL*11/12 contained an additional overlapping SBP-superfamily domain.

### 2.5. Chromosome Localization, Gene Duplication, and Collinearity Analysis

**Figure 4:**
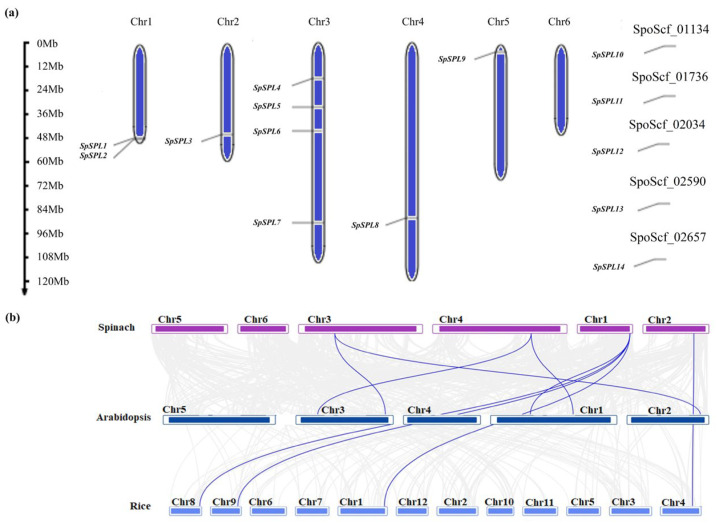
Chromosomal distribution and collinear analysis of *SpSPL* gene members. (**a**) The chromosomal distribution of *SpSPL* genes. The marks on the leaf hand represent the specific locations of *SpSPL* gene members across six chromosomes. Tandem duplications are highlighted in the accompanying table, indicating clusters of *SpSPL* genes that have undergone tandem duplication events; (**b**) the collinear analysis of *SpSPL* genes in spinach and its close relatives. Gray lines in the background represent collinear relationships throughout the genomes of spinach and its close relatives (Arabidopsis and rice). The blue lines primarily denote collinear *SpSPL* gene pairs with Arabidopsis and rice, highlighting conserved synteny and shared evolutionary history among these species.

Based on genomic information, the chromosomal location of all *SpSPL* genes was identified across six chromosomes (Figure 3a). The *SpSPL* genes were renamed according to their chromosomal distribution. Beginning with *SpSPL*1 and *SpSPL*2 on Chr1, followed by *SpSPL*3 on Chr2 and *SpSPL*4/5/6 on Chr3, *SpSPL*7 and *SpSPL*8 were on Chr4 and Chr5, respectively (Table 1; Figure 4a). This systematic gene renaming standardizes the identification, also reflects their physical arrangement on chromosomes, and potentially links to functional relationships. Gene duplication analysis (Table 3) identified *SpSPL*10/13 (segmental duplication, Ka/Ks = 0.3729), *SpSPL*1/2 (tandem duplication, Ka/Ks = 0.2125), and *SpSPL*4/7 (tandem duplication, Ka/Ks = 0.4773) as duplicated gene pairs. In the collinear analysis of spinach SPL genes with Arabidopsis and rice, a conserved synteny relation was revealed (Figure 4b). Blue lines in the collinarity analysis figure indicate collinear SPL gene pairs, emphasizing shared evolutionary history among these species.

### 2.6. Gene Structure Distribution, Cis-Acting Elements, and Transcription Factor Binding Sites in the Promoter Region of SpSPLgene Members

**Figure 5:**
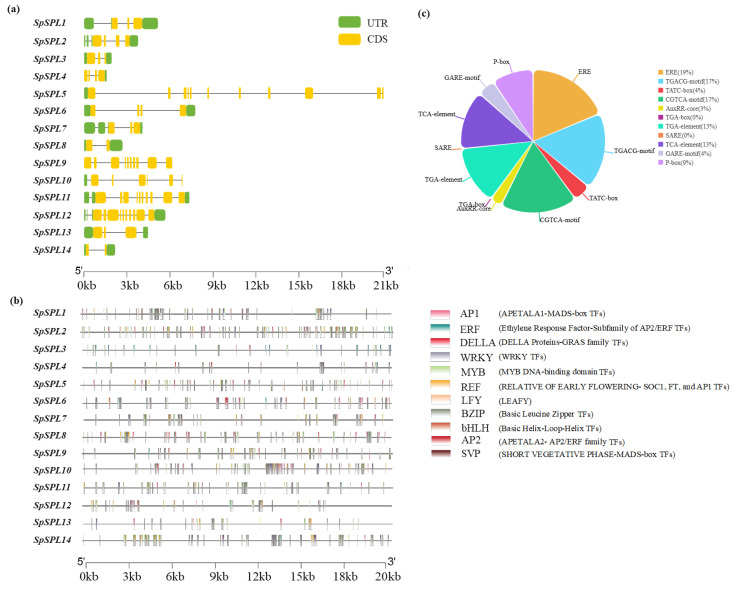
Gene structure distribution, Cis-acting hormone-responsive elements, and transcription factor binding sites in the promoter region of *SpSPL* gene members in spinach. (**a**) Gene structures of *SpSPL* gene members, showing full-length coding sequences. Exons are represented as yellow boxes, and introns are depicted by black lines. The exon–intron structure of *SpSPL* genes was predicted using GSDS, with the scale (0–21 kb) at the bottom indicating the relative size of introns and exons; (**b**) these are transcription factor binding sites in the 2 kb promoter region of *SpSPL* gene members. The binding sites were predicted using “PlantPAN 4.0” based on Position Weight Matrix (PWM) models. Different colored boxes represent distinct transcription factor regulatory motif types, with each color corresponding to a specific class of TFs involved in various biological processes; (**c**) enrichment of phytohormone-related cis-acting elements’ percentage share to the promoter regions of *SpSPL* gene members. TFs, transcription factors; GSDS, gene structure display server.

Gene structure analysis discovered considerable variation in exon–intron organization, and the exons ranged from 2 to more than 10 (Figure 5a). *SpSPL*1/2/3 showed compact gene structures with fewer exons and shorter introns, whereas *SpSPL*9/11/12 showed expanded gene architectures with multiple exons and longer introns. These structural diversities reflect evolutionary divergence and gene duplication events within the spinach SPL gene members. We performed a comprehensive analysis of cis-regulatory elements in the 2000 bp upstream promoter regions to investigate potential hormonal regulation of *SpSPL* genes in spinach. This analysis identified six major hormone-responsive elements. These were abscisic acid (ABRE), ethylene (ERE), methyl jasmonate (MeJA; TGACG-motif, CGTCA-motif, and TATC-box), auxin (AuxRR-core, TGA-box, TGA-element), gibberellin (GARE-motif and P-box), and salicylic acid (SARE, TCA-element). Among the gene family, *SpSPL*9 harbored the highest number of abscisic acid elements (ABRE = 9), suggesting a strong potential to respond to ABA. *SpSPL*8 showed regulation with EREs, two MeJA-related (TGACG- and CGTCA-motif), and two gibberellin-responsive P-boxes. Similarly, *SpSPL*5/4 harbored MeJA, ethylene, and salicylic acid-responsive elements, indicating their role in multiple hormone signaling pathways. *SpSPL*3/2 was enriched in ethylene-responsive elements (ERE), one TGACG-motif (MeJA-responsive), four TGA-elements (auxin-responsive), and one salicylic acid-related TCA-element. *SpSPL*8 shows richness in the cis-element profile containing three ABREs (ABA), three EREs, two MeJA motifs (TGACG and CGTCA), one TGA-element (auxin), and two gibberellin P-boxes (Figure 5c) (Table S2). The analysis revealed a diverse range of cis-acting regulatory elements and transcription factor binding sites associated with floral development and hormonal signaling. Notably, several floral meristem identity regulators were identified, including APETALA1 (AP1), LEAFY (LFY), and SHORT VEGETATIVE PHASE (SVP), which are key components of the floral transition network. The detection of RELATIVE OF EARLY FLOWERING 6 (REF6) binding motifs suggests epigenetic regulation and activation of flowering-related genes such as FT, AP1, and SOC1. Binding sites for MADS-box transcription factors within *SpSPL* promoters suggest that these genes may be directly regulated by MADS-box transcription factors. This indicates that *SpSPL* genes could integrate signals from phase transition by contributing to the precise timing of the vegetative to reproductive phase transition in spinach (Figure 5b).

### 2.7. Expression Profiling of SpSPL Gene Members

**Figure 6:**
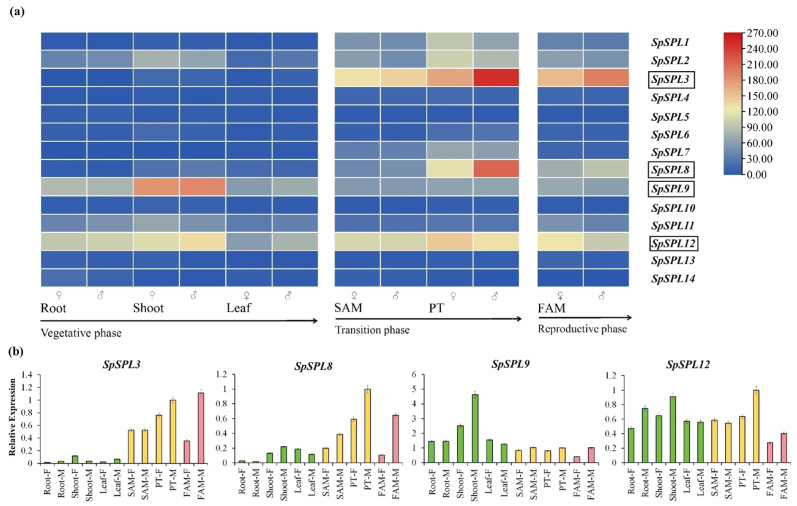
Transcriptomic expression profiling and qRT-PCR validation of *SpSPL* gene members across vegetative, transition, and reproductive stages of dioecious spinach. (**a**) The gene expression heatmap of *SpSPL* gene members. Heatmap showing log2 (TPM + 1) normalized expression of *SpSPL* genes across vegetative, transition, and reproductive stages in male and female spinach. Differential expression was determined using DESeq2 (|log2FC| ≥ 1, FDR < 0.05; *n* = 3 biological replicates per stage). The red and green colors indicate varying expression levels of *SpSPL* genes, from high to low. The tissues cover diverse developmental stages of both male and female plants in dioecious spinach. The table includes Transcripts per Million (TPM) expression values from RNA sequencing, providing a quantitative overview of gene expression patterns across different developmental stages; (**b**) qRT-PCR analysis of *SpSPL*3, *SpSPL*8, *SpSPL*9, and *SpSPL*12 expression at vegetative (root, shoot, leaf) (green bars), transition (SAM, PT) (yellow bars), and reproductive stages (FAM) (pink bars); SAM, shoot apical meristem; PT, phase transition meristem; FAM, flower apical meristem.

Figure 6a shows the expression specificity of *SpSPL* genes across vegetative, transition, and reproductive tissues within transcriptome data of both male and female spinach. The average RNA-seq library size was ~44 million high-quality clean reads, of which 97% mapped to the spinach reference genome, which was SpinachBase v1.0. The mean base quality exceeded 94% Q30. Gene-level TPMs were obtained for all predicted *SpSPL* transcripts, and differential expression was assessed across vegetative, transition, and reproductive stages using DESeq2 (|log2FC| ≥ 1, FDR < 0.05) (Table S3). Heatmaps were generated using log2 (TPM + 1) transformed data to illustrate relative expression patterns, suggesting that different *SpSPL* gene members may play stage- and tissue-specific roles in spinach growth and reproduction. Based on RNA-seq data, *SpSPL*3, *SpSPL*8, *SpSPL*9, and *SpSPL*12 were selected for further analysis due to their distinct developmental stage-specific expression patterns (Table S4). *SpSPL*3 and *SpSPL*8 displayed low expression during the vegetative phase and were significantly up-regulated during the transition and reproductive stages in both male and female spinach tissues. *SpSPL*9 showed consistently high expression in vegetative phase tissues, especially in the shoots of male and female spinach. *SpSPL*12 showed moderate expression in vegetative tissues, peaked in the transition stage, and declined in the reproductive phase of spinach tissues. To validate the RNA-seq findings, qRT-PCR was performed for the selected *SpSPL* genes (*SpSPL*3, *SpSPL*8, *SpSPL*9, and *SpSPL*12) across these three developmental stages (vegetative, transition, and reproductive) in both male and female spinach tissues (Figure 6b). The qRT-PCR result confirmed expression trends observed in RNA-seq TPM values. Specifically, *SpSPL*3 and *SpSPL*8 maintained low transcription levels in vegetative tissues, with a notable peak particularly in PT tissues, and remained high in reproductive stage tissues. *SpSPL*12 showed moderate expression in vegetative tissues with a notable peak during the transition stage, supporting its potential role in phase change. In contrast, *SpSPL*9 demonstrated relatively high expression across all developmental stages of spinach.

### 2.8. Identification of miR156 and Network Analysis in Spinach

**Figure 7:**
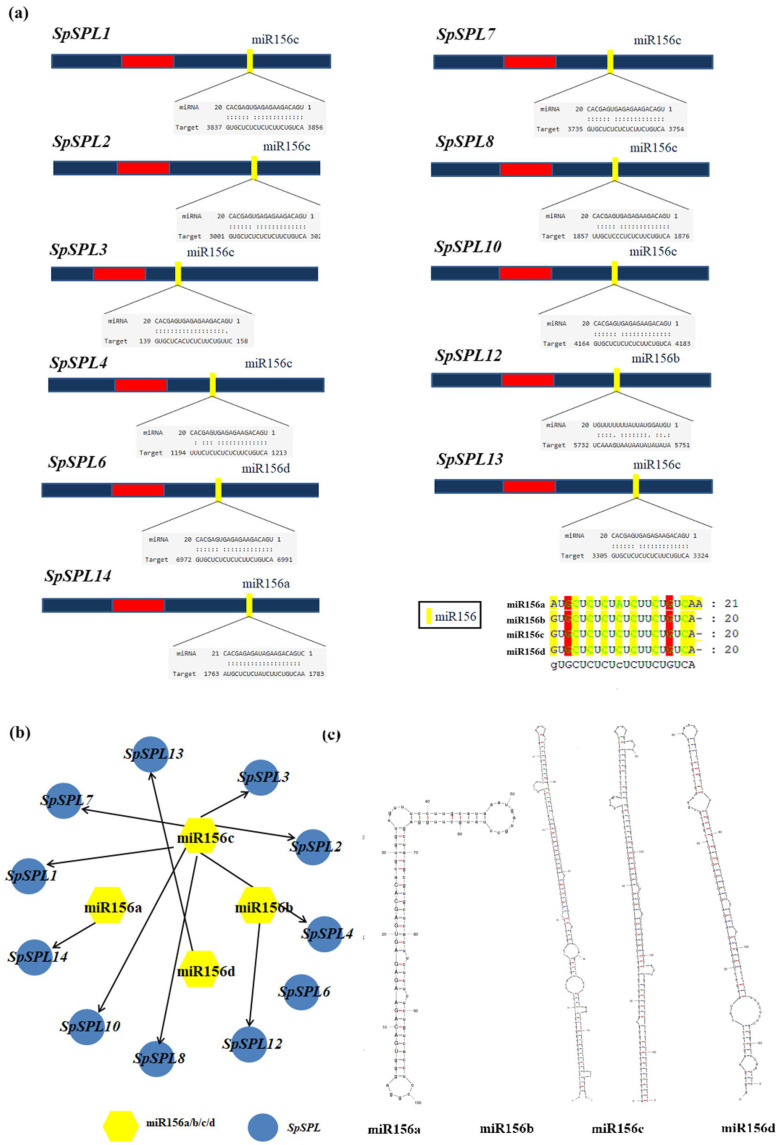
MicroRNA (miR156a/b/c/d) interaction network and sequence analysis of *SpSPL* gene members in spinach. (**a**) The prediction of miRNA target sites (represented by yellow highlights) within the nucleotide sequences of *SpSPL* transcripts. These target sites indicate potential interactions between miR156 and SPL gene members, suggesting their regulatory role in modulating SPL gene expression in spinach. These target sites indicate potential interactions between miR156 and SPL genes, suggesting their regulatory role in modulating SPL gene expression in spinach; (**b**) in silico prediction of miRNA-target interactions shows the potential for miR156a/b/c/d to target specific *SpSPL* genes. (**c**) Predicted secondary structure of the precursor transcript for putative miR156 generated using mfold. Here, mfold refers to the RNA folding web tool commonly used for predicting RNA secondary structures.

To elucidate the regulatory interaction between *SpmiR156* and SPL TFs in spinach, in-silico target prediction was performed. A total of 11 of the 14 identified *SpSPL* genes harbored conserved binding sites for miR156 isoforms, indicating post-transcriptional regulation (Figure 7a). *SpmiR156*c emerged as the predominant isoform of *SpSPL*1/2/3/4/7/8/10/13. Other than this, *SpSPL*12, *SpSPL*14, and *SpSPL*6 were specifically targeted by miR156b, miR156a, and miR156d, respectively. Network visualization of the miRNA156a/b/c/d interaction revealed a hub-like architecture with distinct miR156 isoforms converging on specific subsets of *SpSPL* genes (Figure 7b). Secondary structure prediction of pre-miR156 variants confirms the conserved stem loop hairpin configuration required for miRNA isoforms, while sequence alignment demonstrated high conservation among mature miR156 isoforms in spinach (Figure 7c, Table S5). Collectively, these findings highlight the combinatorial regulatory dynamics of miR156 in modulating SPL gene expression during the vegetative to reproductive phase transition in dioecious spinach.

### 2.9. Gene Ontology (GO) Enrichment Analysis

GO enrichment analysis indicated that spinach SPL genes are predominantly involved in developmental regulation, including vegetative phase transition and floral phase development (Figure S2). Molecular function terms were enriched for DNA-binding and cis-regulatory region binding, while cellular component analysis indicated nucleus localization. Further enrichment analysis based on protein domains and local network clusters identified conserved SBP domains and suggested interaction with floral regulatory modules (Figure S3). UniProt keyword analysis highlighted transcriptional regulation and differentiation with DNA-binding TF activity and zinc finger motifs as a prominent term, reflecting the canonical roles of SPL proteins. The top 25 GO enrichment terms (Figure S4) highlight strong enrichment in nuclear, nucleoplasm, and intracellular organelles (e.g., membrane-bounded organelles), underscoring roles in transcriptional control and cellular organization. These findings imply conserved functions in transcriptional control, and compartmentalized cellular activities covered the involvement of *SpSPL* genes in key developmental transitions, particularly in pathways associated with phase transition, flower morphogenesis, and meristem identity. Collectively, these findings emphasize the conserved and multifaceted regulatory roles of *SpSPL* genes in regulating developmental phase transitions, meristem identity, and flower morphogenesis in spinach.

### 2.10. Proposed Model of miR156-Mediated Regulation of SpSPL Genes

**Figure 8:**
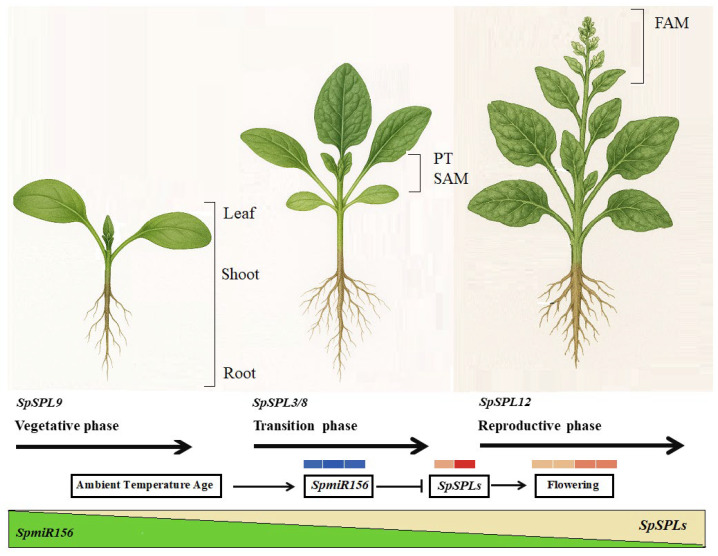
A proposed model depicting the regulatory role of the *SpmiR156-SpSPL* module in dioecious spinach at ambient temperatures. At ambient temperatures (22 °C), the expression of *SpmiR156* is high, and low *SpSPL* protein abundance maintains the plant in the vegetative phase, delaying reproductive transition. As miR156 expression declines, this attenuation of *SpmiR156*-mediated repression allows for the accumulation of *SpSPL* proteins. SPLs then activate the expression of genes promoting reproductive phase identity. This model positions *SpmiR156* as a central component for the vegetative to reproductive phase transition.

The proposed model depicts how miR156 modulates the vegetative to reproductive phase transition in dioecious spinach under ambient temperature conditions (Figure 8). During the early vegetative stage, high *SpmiR156* represses *SpSPL* genes, thereby maintaining vegetative growth and delaying reproductive phase initiation. As the plant ages, the progressive decline in miR156 abundance alleviates this repression, leading to the transcriptional activation of SPL genes. In accordance with the decline in miR156, *SpSPL*3 and *SpSPL*8 exhibit low expression during the vegetative stage but are markedly up-regulated during the transition phase. This temporal pattern supports their regulation by miR156 and implicates them in initiating reproductive development. Together, the model provides systematic insight into how the age-dependent attenuation of miR156 enables *SpSPL-*mediated transcriptional reprogramming, thereby establishing reproductive competence in spinach.

## 3. Discussion

### 3.1. An Overview of SPL Gene Family Members

SPL TFs are family members with a highly conserved SBP domain of photosynthetic plants [20]. In recent years, SPL gene family have been identified in in various plant, such as 17 members in Arabidopsis (*Arabidopsis thaliana*) [24], 19 in rice (*Oryza sativa*) [32], 22 in green-pea (*Pisum sativum* [35], 24 in oil palm (*Elaeis gineensis*) [36], 19 in blue horn (*Catalpa bungei*) [37], 16 in red pepper (*Capsium Annuum*) [38], 18 in grape (*Vitis vinifera*) [39], 15 in pomegranate (*Punica granatum*) [40], and 56 in princess tree (*Paulowinia tomentosa*) [25]. The SBP domain is a distinctive class of TFs exclusive to the plant kingdom and is critical in various processes of juvenile (vegetative) to adult (reproductive) phase transition in plants [25,41]. However, to date, no information is available on the identification and characterization of SPL genes in spinach at the vegetative to reproductive phase transition. In this study, we identified 14 *SpSPL* members (Table 1) in the spinach genome through a bioinformatics approach and dissected their expression by analyzing RNA-seq. data from vegetative, transition, and reproductive stage tissues. The fourteen SPL members we identified in spinach are comparatively fewer than the SPL members identified in other plant species. Present information on spinach *SpSPL* explained the evolutionary dynamics and functional diversity of this gene family. Phylogenetic analysis highlighted sequence similarity with Arabidopsis, rice, oat, and oil palm (Figure 2), suggesting conserved functional roles across species. Our findings are consistent with previous reports on the identification of SPL genes in Arabidopsis [24], rice [32], and oil palm [36]. The SPL genes in spinach were identified through the spinach genome feature-conserved SBP domain “PF03110”, which is essential for DNA binding and regulating downstream target genes. The SBP domain is considered a key feature in the SPL gene family in Arabidopsis and rice [42]. Structural analysis indicated conserved exon–intron organization, consistent with functional conservation (Figure 3). SBP domain motifs were found in all the identified *SpSPL* proteins. Conserved motif analysis identified 10 motifs in spinach SPL genes (Figure 3a), as reported in other plants, including foxtail millet (*Setaria italic*) [43], alfalfa (*Medicago sativa*) [44], and quinoa (*Chenopodium quinoa*) [45]. Chromosomal mapping indicated genes spread across multiple chromosomes (Chr1 to Chr5) (Figure 4), with gene duplication events contributing to the expansion of the family. Ka/Ks ratio analysis suggested that genes underwent purifying selection, highlighting their crucial functional roles in plant development (Table 3). The uneven distribution of 14 *SpSPL* genes on chromosomes also coincides with the previous reports on SPL gene family distribution in alfalfa [46] and foxtail millet genomes [43]. Tandem and segmental duplications are the primary causes of gene family expansion and functional diversity [47]. Our findings emphasize that the PF03110 domain serves as a key molecular signature for this family, which has been implicated in regulating vegetative to reproductive transitions in spinach. Understanding the genomic and structural diversity of this gene family provides insights into their roles in controlling phase transitions in spinach and also in other plants.

### 3.2. SpmiR156-SpSPL Module in Phase Transition

An important result emerging in this study is the identification of *SpmiR156* as a key regulator of SPL genes in spinach. The majority of SPL gene members harbor complementary regions to miR156, and the *SpmiR156*-*SpSPL* molecular module is established as a key regulator in various plant developmental processes [12,48]. A total of 11 out of 14 identified *SpSPL* genes contain conserved miR156 isoforms. The predicted miR156 binding sites are located within the nucleotide sequences of the SPL genes, mostly in the coding regions, indicating potential interactions between *SpmiR156* and *SpSPL* genes (Figure 6a). Previous reports on *Jatropha curcas* [49], *Glycine max* [50], *Medicago truncatula* [44], and *Brassica juncea* [51] SPL genes demonstrate the conservation of miR156-mediated post-transcriptional regulation. The post-transcriptional regulation of SPL genes by miR156 determines fine tuning the functional specificity and temporal expression of SPL TFs [14]. Specifically, *miR156a/b/c/d* was shown to mediate the regulation of these genes across various developmental stages in spinach (Figure 7). This implies that *SpmiR156* isomers may constitute a complex regulatory network in spinach, contributing to the modulation of diverse biological processes. This hypothesis is supported by seasonal growth [40], vegetative to reproductive phase change, and photosynthesis [52]. The expression dynamics of *SpSPL* genes across vegetative, transition, and reproductive phase tissues identified an inverse relationship between *SpmiR156* levels. The *miR156-SPL/SBP* module regulates, in part, the developmental phase transitions that have been confirmed in tomato [53], potato [54], and alfalfa [55]. During the vegetative phase, high miR156 levels effectively suppressed SPL domain gene expression, maintaining the plant in a juvenile vegetative state. As the plant ages and transitions into the reproductive phase, miR156 expression decreases, which allows for the up-regulation of SPL genes. In Arabidopsis, *SPL3* and *SPL9* are miR156-targeted genes that directly activate the expression of floral induction genes [56,57]. Similarly, genes like *SpSPL*3 and *SpSPL*8 showed significant up-regulation during the phase transition stage tissues, which was preceded by *SpmiR156*-mediated repression during the juvenile vegetative stage (Figure 8). These results were further validated by qRT-PCR (Figure 6), which confirmed the RNA-seq data trend and provided strong evidence for the *SpmiR156*-mediated regulation of *SpSPL* genes. Our data suggest that the *SpmiR156*-*SpSPL* regulatory module plays a pivotal role in the timing of vegetative to reproductive phase transition in spinach, with implications for controlling developmental phase shifts in plants.

Although our prediction of *SpmiR156-SpSPL* interactions is based on computational analysis, the regulatory module has been experimentally validated in several plant species. For example, degradome sequencing confirmed the miR156-guided cleavage of SPL transcripts in *Jatropha curcas*. In Jatropha, there was significant variability in the morphology of leaves of 1- and 12-month-old shoots of plants. The expression of *JcSPL3* increased significantly in leaves from 12-month-old plants compared to that from 1-month-old plants. Together, these strongly suggest that *JcSPL3* may be responsible for the vegetative phase transition in Jatropha. This was confirmed by the overexpression of *JcSPL3* into Arabidopsis, which revealed an earlier flowering phenotype [49]. In *Malus sieversii*, 5′-RACE and dual luciferase assays verified that *miR156ab* targets and cleaves the *MsSPL13* transcript, establishing a functional miR156-SPL regulatory module that enhances drought tolerance through the modulation of auxin metabolism [58]. Further studies in populus have provided strong experimental evidence supporting the conserved regulatory function of the miR156-SPL module. The overexpression of *MIR156k* (35S::MIR156k) led to the strong repression of *SPL* genes, validating a conserved miR156-SPL regulatory module that governs vegetative to reproductive phase transition [25]. This evidence supports the evolutionary conservation of the *SpmiR156-SpSPL* network and reinforces its likely role in phase change regulation in spinach.

### 3.3. Functional Implication in Dioecious Spinach

The functional implications of *SpSPL* genes are further highlighted by their involvement in hormonal signaling (Figure 5). A total of 10 hormone-responsive cis-elements in 2000 bp upstream promoter regions of *SpSPL* genes were identified, particularly ethylene, abscisic acid (ABA), methyl-jasmonate (MeJA), and auxin (IAA) elements, suggesting that these genes are regulated by multiple hormonal pathways. This aligns with the findings in apple (*Malus domestica*), where many *MdSBP* genes exhibited up-/down-regulation in response to ethylene, MeJA, and ABA [59]. These elements play a role in modulating SPL gene expression in response to developmental processes. GO enrichment analysis exhibited that *SpSPL* genes are associated with flower meristem identity and cellular signaling, reinforcing their role in the vegetative to reproductive phase transition. This is consistent with studies in Arabidopsis, where SPL genes (e.g., *SPL3* and *SPL9*) induce flowering through the activation of floral integrator genes like LEAFY (*LFY*) and APETALA1 (*AP1*) [57,60,61]. A key finding is the potential to manipulate the *SpmiR156*-*SpSPL* module to control phase transition in spinach (Figure 8). In many plants, miR156 suppresses SPL genes during the juvenile phase, and its decline permits SPL-mediated flowering [19]. Modulating this module could adjust flowering onset, presenting opportunities for spinach breeding programs.

## 4. Materials and Methods

### 4.1. Plant Material

Spinach (*Spinacia oleracea*) germplasm II9A0075 (A75) were obtained from the Institute of Crop Sciences, Chinese Academy of Agricultural Sciences, Beijing, China. Seeds were surface sterilized with 10% *v*/*v* sodium hypochlorite for 5 min [62] and grown at 22 °C for a 16/8 h photoperiod in the Fujian Provincial Key Laboratory, Haixia Applied Plant System Biology Laboratory, Fuzhou, China (Date: 10 October 2024) [63]. Tissues, including the root, shoot, leaves, Stem Apical Meristem (SAM), Phase Transition meristem (PT), and Flower Apical Meristem (FAM), were collected from male and female spinach plants. Collected samples were immediately frozen in liquid nitrogen and stored at −80 °C for RNA extraction and gene expression analysis. Raw transcriptome data files of these developmental stages of Female (F) and Male (M) plants were used.

### 4.2. RNA Extraction, Library Construction, and Sequencing

Total RNA was extracted from spinach tissues using TRIzol reagent (Invitrogen, Waltham, MA, USA), and RNA quality was assessed with NanoDrop and agarose gel electrophoresis. Libraries were prepared with the NEBNext^®^ Ultra™ RNA Library Prep Kit (NEB, Ipswich, MA, USA) and sequenced on the Illumina NovaSeq 6000 platform to generate paired-end reads (PE150).

### 4.3. RNA-Seq Data Analysis

Raw RNA-seq reads were quality-checked using “FastQC v0.12.1” and low-quality reads were removed using “Trimmomatic v0.39”; clean reads were mapped to the reference spinach genome (Sp75 genome) (http://spinachbase.org/ftp/genome/Sp75/ accessed on 15 December 2024) [34] using “STAR aligner”. Mapping reads referring to each transcript were assembled, and Transcript per Million (TPM) values were calculated using the expression quantifier “StringTie”. The dynamic expression pattern was presented using a heat map based TPM numeric values of different samples using the “HeatMap Illustrator” function in TBtools (v2.119). Differentially Expressed Genes (DEGs) were identified with “DESeq2 v1.36.0” using the criteria |log2FoldChange| ≥ 1 and adjusted *p*-value (FDR) < 0.05. Three biological replicates were used per condition. Statistical significance and normalization were applied across replicates before generating heatmaps. Expression pattern comparisons were made between the two sex types (F, female; M, male) and up-/down-regulating genes were visualized by the online platform “OmicShare” (https://www.omicshare.com/tools/ accessed on 12 March 2025).

### 4.4. Identification and Physicochemical Properties Analysis of SpSPL Gene Members

The spinach genome (Sp75 genome; released 2021) (http://spinachbase.org/ftp/genome/Sp75/ accessed on 15 May 2025) [34] served as the foundation for this research, enabling us to identify and analyze SPL genes. In total, 16 Arabidopsis SPL protein sequences were downloaded (https://www.Arabidopsis.org/ accessed on 15 May 2025). Spinach genome sequences were blasted with Arabidopsis SPL protein sequences. The Hidden Markov Model (HMM) of the SBP domain “PF03110” was downloaded from the Pfam database (http://pfam.xfam.org/ accessed on 20 May 2025), and the spinach was searched using HMMER 3.0 software. The results from both methods were merged. For further screening, the SMART online site (https://smart.embl.de/ accessed on 20 May 2025) and the CDD database (https://www.ncbi.nlm.nih.gov/cdd accessed on 20 May 2025) were used. Ultimately, 14 SPL/SPB gene members were obtained for further analysis. ExPASY (https://www.expasy.org/ accessed on 22 May 2025) was used, and physicochemical properties of the SPL box proteins were analyzed, such as CDS length, amino acid length (AA), molecular weight (KDa), isoelectric point (PI), and GRAVY values. SPL box proteins’ subcellular localization was predicted using CELLO v2.5 (http://cello.life.nctu.edu.tw/ accessed on 22 May 2025) and WoLF-PSORT (http://www.genscript.com/wolf-psort.html accessed on 22 May 2025).

### 4.5. Phylogenetic and Sequence Analysis of SpSPL Gene Members

To understand phylogenetic relationships, multiple protein sequence alignment was performed based on *Arabidopsis thaliana*, *Avena sativa*, *Oryza sativa*, and *Elaeis guineensis SPL* gene members, with 14 full-length *Spinacia oleracea SPL* gene members using MEGA 11 software with default parameters. The resulting tree Newick file was downloaded, and the online beautification tool iTOL (https://itol.embl.de/ accessed on 25 May 2025) was used. The same method was repeated to build phylogenetic relationships with known function members of *Arabidopsis thaliana* and *Oryza sativa*, with 14 SPL genes of *Spinacia oleracea*. *SPL* genes of *Spinacia oleracea* were analyzed by the online MEME tool (https://meme-suite.org/ accessed on 25 May 2025) to analyze conserved motifs (number of motifs set to 10) of the *SpSPL* protein. TBtools software (v2.119) was used to visualize conserved domains.

### 4.6. Chromosome Distribution, Collinearity Analysis, and Gene Duplication of SpSPL Gene Members

Gene location was visualized using the “Gene Structure View” function in TBtools (v2.119) of *SpSPL* box family members in *Spinacia oleracea* and named according to the reference genome (Sp75 genome) (http://www.spinachbase.org/ accessed on 30 May 2025). Collinearity analysis with *Spinacia oleracea* was performed using “One Step MCScanX” in TBtool (v2.119) with default parameters. The genome data of *Arabidopsis thaliana*, *Oryza sativa*, *Chenopodium quinoa*, and Beta vulgaris were downloaded from the NCBI database (https://www.ncbi.nlm.nih.gov/ accessed on 20 May 2025). Tandem and segmental duplication were calculated by identifying duplicated genes through a Blast search with TBtools (v2.119), using identity > 80% and query coverage > 70%. The number for synonymous substitutions per synonymous site (Ks) and non-synonymous substitutions per non-synonymous site (Ka) were acquired using the straightforward Ka/Ks calculator within TBtools software. Subsequently, the Ks values acquired for each pair of genes were converted into divergence times as “T = Ks/2λ, with λ = 6.5 × 10^−935^”. This calculation was based on an assumed substitution rate (λ) of 6.1 × 10^−9^ substitutions per site per year [64].

### 4.7. Multi-Sequence Alignment and Domain Confirmation of SpSPL Gene Members

Multi-sequence alignment and domain analysis were performed using the MEGA11 software. Full-length SPL member protein sequences from *S. oleracea* were aligned by ClustalW with default parameters. Full-length amino acid sequences of SPL proteins were submitted to MEME Suite v5.5.0 (Multiple Expectation Maximization for Motif Elicitation). The MEME output revealed a motif, which includes two zinc finger-like structures (Cys-Cys-His-Cys and Cys-Cys-Cys-His) and a nuclear localization signal (NLS). To generate the motif signature, the “Visulize Motif Pattern” function in TBtools (v2.119) was used.

### 4.8. Gene Structure and Cis-Element and Transcription Factor Binding Sites in Promoter Region

Gene structures were analyzed using the “Visualize Gene Structure” function in TBtools (v2.119). For cis-element promoter analysis, the Gff3-file of reference genome version SpinachBase v1.0 was used to extract a promoter region of 2-kb of the translation start site, ATG of the 14 SPL member genes, using strand-specific coordinates, ensuring no overlap with upstream coding sequences. Identified promoter sequences were scanned for cis-acting regulatory elements using “PlantPAN 4.0” (https://plantpan.itps.ncku.edu.tw/plantpan4/ accessed on 10 October 2025). Default significance thresholds (*p*-value ≤ 1 × 10^−4^) were applied to identify high-confidence TF binding sites. All regulatory elements were systematically annotated and visualized using TBtools (v2.119).

### 4.9. MicroRNAs Identification and Analysis of SpSPL Gene Members

Mature miRNAs were obtained online from “miRBase” (http://www.mirbase.org/ accessed on 2 June 2025). *SpSPL* genes targeted by miR156 were predicted by searching the coding sequence regions of 14 SPLs for complementary sequences online for “psRNATarget” (https://www.zhaolab.org/psRNATarget/ accessed on 2 June 2025). For miR156a/b/c/d secondary structure prediction, miR156 members were queried and downloaded from the “miRBase” database (http://www.mirbase.org/ accessed on 2 June 2025). The secondary structures of *miR156a/b/c/d* were predicted using the online tool “RNA Folding FORM V2.3” (http://www.unafold.org/mfold/ accessed on 3 June 2025) with default parameters. “Clustal Omega” (http://www.clustal.org/omega/ accessed on 3 June 2025) was employed for the multiple sequence alignment of the mature miR156 sequences.

### 4.10. qRT-PCR Validation of the Expression of Genes

Total RNA from vegetative, transition, and reproductive stage tissues of spinach was extracted using the “RNeasy Plant Mini Kit” (QIA GEN, Hilden, Germany). Approximately 1 μg of total RNA was used for reverse transcription (cDNA synthesis) using the “HiScript^®^ II 1st Strand-cDNA Synthesis Kit (+gDNA wiper) (Vazyme, Nanjing, China). Quantitative real-time PCR (qRT-PCR) was performed with the “TB Green^®^ Premix Ex-Taq™ II” (Tli RNaseH Plus) kit (TaKaRa, Dalian, China). Gene expression was normalized using the “2^−ΔΔCT^ method”, with ACTIN2 EF1a as the internal reference for spinach. Expression levels of *SpSPL*3, 8, 9, and 12 were measured, and primer sequences are listed in Table S6.

## 5. Conclusions

This study provides the first comprehensive genome-wide characterization of the SPL TFs family in spinach and demonstrates their regulation by miR156 during developmental phase transitions. Our findings reveal that *miR156c* represses *SpSPL3* and *SpSPL8* during the vegetative stage, while its gradual decline in later stages permits SPL activation, thereby facilitating vegetative to reproductive phase transition. Together with gene ontology and cis-element analyses, these results highlight the central role of the *SpmiR156*-*SpSPL* regulatory module in integrating hormone signaling with reproductive development in spinach.

## Figures and Tables

**Table 1:** Genomic characteristics of identified *SpSPL* family gene members in spinach.

Gene IDs	Putative IDs	CHR	Type	CDS Length	Protein Length	Start	End	Strand	Introns	Exons	Subcellular Localization
*Spo*04944	*SpSPL1*	chr1	gene	1185	394	48,064,777	48,070,072	+	2	3	nucleus
*Spo*04935	*SpSPL2*	chr1	gene	1362	453	48,289,996	48,293,867	+	3	4	nucleus
*Spo*08630	*SpSPL3*	chr2	gene	858	285	46,729,688	46,731,659	+	2	3	nucleus
*Spo*01383	*SpSPL4*	chr3	gene	948	315	18,256,316	18,257,931	+	4	3	nucleus
*Spo*06804	*SpSPL5*	Chr3	gene	2469	822	19,484,558	19,506,030	+	10	11	nucleus
*Spo*16305	*SpSPL6*	chr3	gene	1092	363	33,003,448	33,011,420	+	3	4	nucleus
*Spo*06968	*SpSPL7*	chr3	gene	1050	349	92,766,316	92,770,496	+	2	3	nucleus
*Spo*26325	*SpSPL8*	chr4	gene	675	224	90,130,430	90,133,186	+	1	2	nucleus
*Spo*02184	*SpSPL9*	chr5	gene	2984	994	3,852,598	3,858,915	−	9	10	plasma membrane
*Spo*06850	*SpSPL10*	*Spo*Scf_01134	gene	1482	493	112,527	119,578	+	5	6	nucleus
*Spo*14961	*SpSPL11*	*Spo*Scf_01736	gene	2970	989	5796	13,340	+	9	10	endomembrane
*Spo*24998	*SpSPL12*	*Spo*Scf_02034	gene	3237	1078	4996	10,827	+	9	10	plasma membrane
*Spo*16283	*SpSPL13*	*Spo*Scf_02590	gene	1563	520	28,195	32,774	+	2	3	nucleus
*Spo*22151	*SpSPL14*	*Spo*Scf_02657	gene	330	109	7708	9930	+	1	2	nucleus

**Table 2:** Physicochemical properties of genome-wide identified *SpSPL* family gene members in spinach.

Gene IDs	Putative IDs	Formulas	Molecular Weight (Da)	Theoretical pI	Instability Index	Aliphatic Index	Gravy
*Spo*04944	*SpSPL1*	C1862H2921N565O595S14	43,190.94	9.23	46.65	58.65	−0.738
*Spo*04935	*SpSPL2*	C2139H3358N630O694S18	49,581.08	8.49	56.9	60.93	−0.616
*Spo*08630	*SpSPL3*	C1391H2160N438O431S14	32,363.97	9.3	75.8	56.49	−0.833
*Spo*01383	*SpSPL4*	C1495H2358N454O481S17	34,932.94	8.57	70.59	63.46	−0.609
*Spo*06804	*SpSPL5*	C4027H6371N1137O1225S53	92,013.95	6.61	47.93	79.29	−0.353
*Spo*16305	*SpSPL6*	C1631H2533N501O548S20	38,569.28	7.11	62.46	52.59	−0.647
*Spo*06968	*SpSPL7*	C1632H2555N493O523S29	38,523.96	7.93	57.67	51.92	−0.64
*Spo*26325	*SpSPL8*	C1035H1698N338O344S15	24,861.83	9.59	66.92	45.85	−0.983
*Spo*02184	*SpSPL9*	C4821H7583N1389O1510S47	110,669.46	5.67	50.26	79.94	−0.419
*Spo*06850	*SpSPL10*	C2406H3791N67O768S26	55,183.14	6.15	54.53	74.14	−0.468
*Spo*14961	*SpSPL11*	C4797H7634N1378O1468S49	109,670.67	7.51	51.33	81.31	−0.391
*Spo*24998	*SpSPL12*	C5119H8107N1497O1620S57	118,370.16	8.13	57.63	71.45	−0.438
*Spo*16283	*SpSPL13*	C2452H3850N712O823S24	57,241.26	5.99	53.83	65.79	−0.582
*Spo*22151	*SpSPL14*	C428H818N174O172S8	12,611.85	7.68	67.45	42.02	−1.093

**Table 3:** Ka, Ks, and Ka/Ks ratios, divergence rates, and duplication types of paralogous *SpSPL* gene pairs in spinach.

Duplicate Gene Pairs Putative IDs	Ka	Ks	Ka/Ks	Duplication	Type of Mutation/Evolution
*SpSPL10/SpSPL13*	0.598279	1.604524	0.37286998	SD	Negitive mutation/Purifing
*SpSPL1/SpSPL2*	0.635706	2.991455	0.212507439	TD	Negitive mutation/Purifing
*SpSPL4/SpSPL7*	0.166343	0.348476	0.477344591	TD	Negitive mutation/Purifing

## Data Availability

The original data presented in this study are publicly available. RNA seq data were deposited at the NCBI under https://www.ncbi.nlm.nih.gov/bioproject/PRJNA1321129, accessed on 10 September 2025.

## Supplementary Materials

The following supporting information can be downloaded at: https://www.mdpi.com/article/10.3390/plants14223543/s1, Figure S1: Multi sequence and domain analysis of spinach *SpSPL* gene members. (a) Multi-sequence alignment of *SpSPL* gene members, highlighting the conserved SBP domain. The black boxes represent the conserved SBP domain, while the Nuclear Localization Signal (NLS) and two conserved zinc finger structures (Zn-1, Zn-2) are shown; (b) Sequence logo representation of Spinach SBP domain. The total height of each stack denotes the degree of conservation at each amino acid position, with the height of each letter inside the stack indicating the relative frequency of related amino acids at that position; Figure S2: Biological, molecular and cellular enrichment analysis of *SpSPL* gene members in spinach. The analysis identifies significant overrepresentation of terms related to floral development, DNA-binding activity, and nuclear localization. Show enriched terms in three Gene Ontology (GO) categories. (a) Biological Process; (b) Molecular Function, and: (B) Cellular Component. The size of each dot represents the number of genes associated with the term (Gene count), and the color intensity corresponds to the statistical significance (False Discovery Rate, FDR). The analysis conclusively shows that the gene set is highly enriched for DNA-binding transcription factors localized to the nucleus, which function as key regulators of specific processes in flower and anther development; Figure S3: Protein domain, local network cluster and annotated keywords enrichment analysis of *SpSPL* gene members in spinach. The analysis was performed using three complementary approaches to identify overrepresented biological themes. (a) Protein Domains and Features (InterPro) enrichment. Shows significant enrichment of DNA-binding domains characteristic of key plant transcription factor families, including the SBP (Squamosa Promoter-Binding) and MADS-box families. (b) Local Network Cluster (STRING) enrichment. Depicting functional clusters from protein-protein interaction networks. The size of the data points corresponds to the number of genes in the cluster, and the color represents the False Discovery Rate (FDR). Key enriched biological processes are labeled, with the most significant terms relating to flower development, initiation, and meristem identity; (c) Annotated Keywords (UniProt) enrichment. The chart summarizes enriched functional keywords. The dot plot shows keywords related to biological processes (e.g., Flowering, Differentiation), protein function (e.g., Developmental protein, DNA-binding), and subcellular localization (e.g., Nucleus). Dot size indicates the number of genes, and color represents the statistical significance (−log10 FDR). Collectively, these analyses robustly indicate that the gene set is highly enriched for transcription factors, particularly from the SBP and MADS-box families, that regulate flowering and floral organ development. FDR, False Discovery Rate; Figure S4: Top 25 GO-enrichment analyses of *SpSPL* gene members in spinach. The bubble chart displays the top 25 significantly enriched GO terms. The Rich Factor (the proportion of genes in the target set associated with the term) is plotted against the significance level −log10 (*p*-value). The size of each bubble corresponds to the number of genes annotated with the specific GO term (GeneNumber), and the color gradient represents the *p*-value, with darker shades indicating greater statistical significance. The analysis shows a strong and significant enrichment for terms related to the nucleus (e.g., nucleoplasm, nuclear speck, nuclear body) and other intracellular membrane-bounded organelles, indicating that the encoded proteins are predominantly localized to these cellular compartments; Table S1: *SpSPL* Transcription Factor Protein Sequences in Spinach with Reference Genome and Putative Gene Annotations; Table S2: Phytohormone-Related Cis-Elements Found in *SpSPL* Genes in Spinach; Table S3: HISAT2 Alignment Statistics of RNA-Seq Reads from Spinach (Sp75 Genome Assembly); Table S4: Transcriptome Expression (TPM: Transcripts Per Million) values of *SpSPL* Genes in Spinach across Vegetative, Transition, and Reproductive Phases; Table S5: MicroRNA (miR156) Types and Nucleotide Sequences Targeting *SpSPL* Genes in Spinach; Table S6: Quantitative Real-Time PCR (qRT-PCR) Primer Sequences for Spinach *SpSPL* Transcription Factor Genes.

## Abbreviations

The following abbreviations are used in this manuscript:

*Sp*	*Spinacia olaracea*
SPL	SQUAMOSA Promoter-Binding Protein-Like
miRNA	MicroRNA
TFs	Transcription Factors
GO	Gene Ontology
qRT-PCR	Quantitative Real-Time Polymerase Chain Reaction
SAM	Shoot Apical Meristem
FAM	Flower Apical Meristem
IM	Inflorescence Meristem
PT	Phase Transition Meristem
F	Female
M	Male
DEGs	Differentially Expressed Genes
CDs	Coding Sequences
IPs	Predicted Isoelectric
GRAVY	Grand Average of Hydropathy
TPM	Transcripts per Million
GSDS	Gene Structure Display Server
CDD	Conserved Domain Database
PWM	Position Weight Matrix
