# Supplementary material for: Transcriptome-Based miR156-Mediated Expression Dynamics of SPL Transcription Factors During Vegetative to Reproductive Transition in Spinach
Source: Plants (Basel). 2025 Nov 20;14(22):3543. doi: 10.3390/plants14223543 (PMC12656122; doi:10.3390/plants14223543)
Supplement: Supplementary file 1 [file plants-14-03543-s001.zip › Supplementary Figures.pdf]

Supplementary Materials

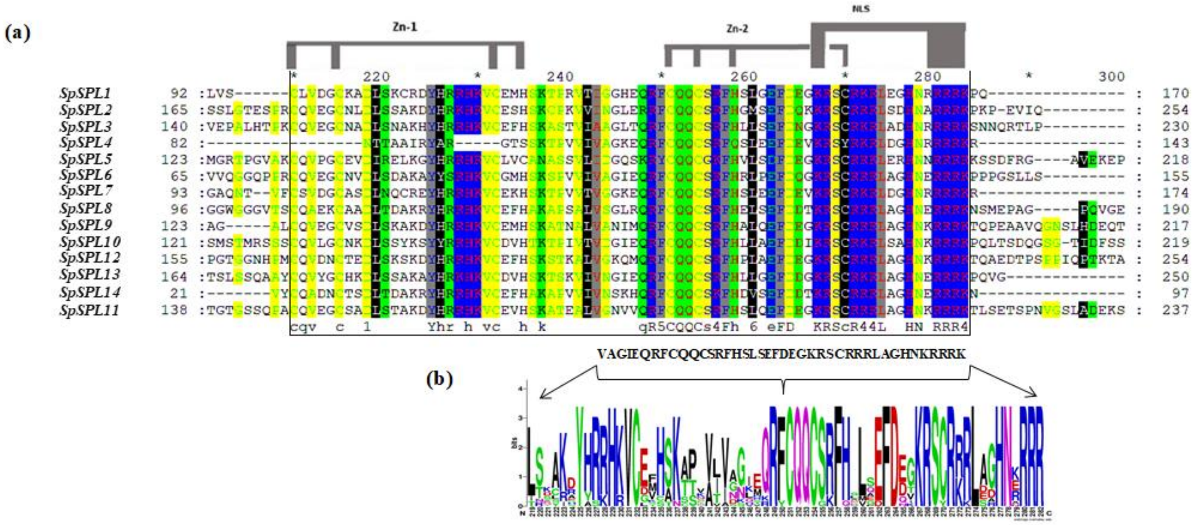

Figure S1. Multi sequence and domain analysis of spinach *SpSPL* gene members. (**a**) Multi-sequence alignment of *SpSPL* gene members, highlighting the conserved SBP domain. The black boxes represent the conserved SBP domain, while the Nuclear Localization Signal (NLS) and two conserved zinc finger structures (Zn-1, Zn-2) are shown; (**b**) Sequence logo representation of Spinach SBP domain. The total height of each stack denotes the degree of conservation at each amino acid position, with the height of each letter inside the stack indicating the relative frequency of related amino acids at that position.

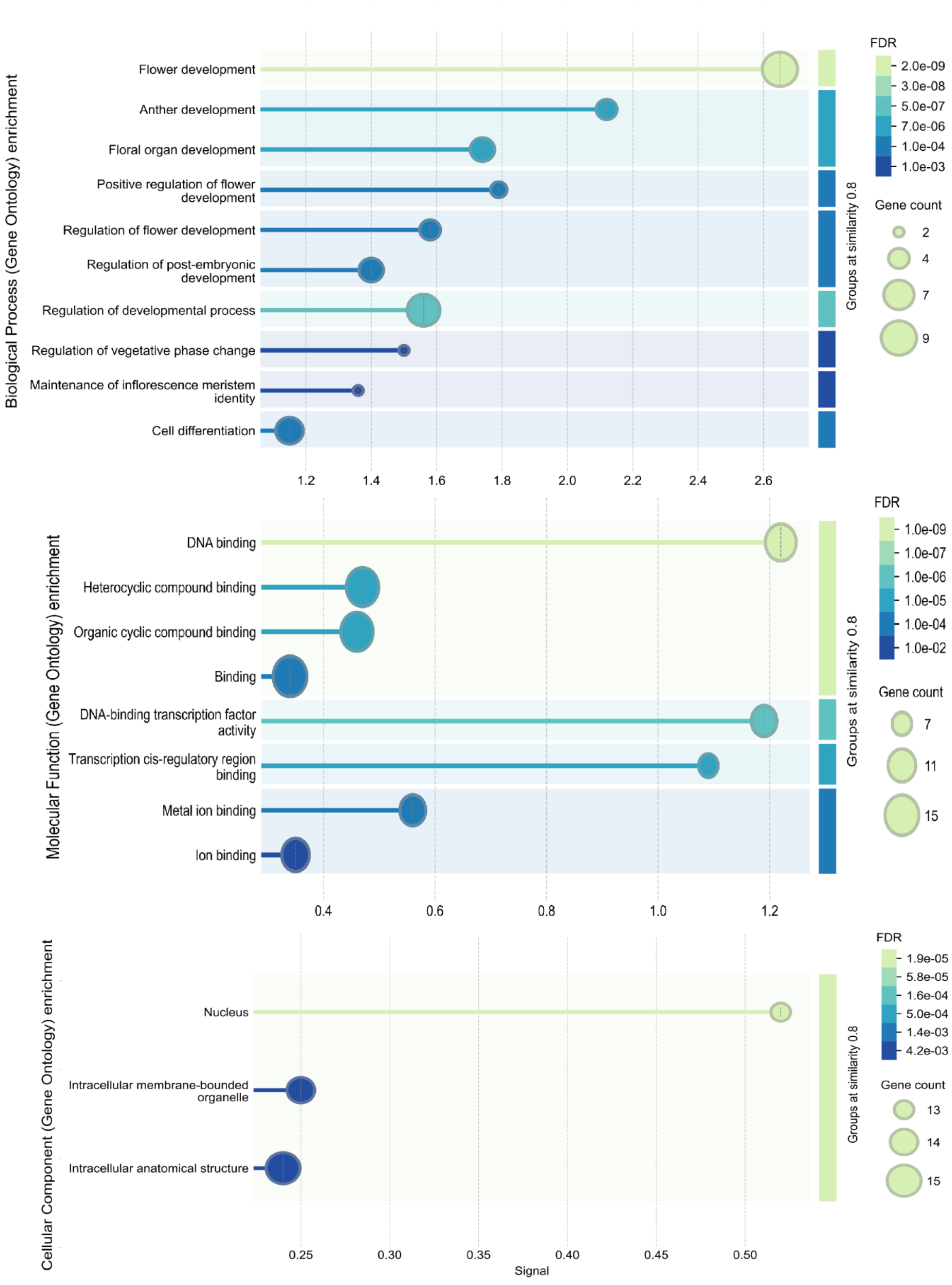

Figure S2. Biological, molecular and cellular enrichment analysis of *SpSPL* gene members in spinach. The analysis identifies significant overrepresentation of terms related to floral development, DNA-binding activity, and nuclear

localization. Show enriched terms in three Gene Ontology (GO) categories. (**a**) Biological Process; (**b**) Molecular Function, and: (B) Cellular Component. The size of each dot represents the number of genes associated with the term (Gene count), and the color intensity corresponds to the statistical significance (False Discovery Rate, FDR). The analysis conclusively shows that the gene set is highly enriched for DNA-binding transcription factors localized to the nucleus, which function as key regulators of specific processes in flower and anther development.

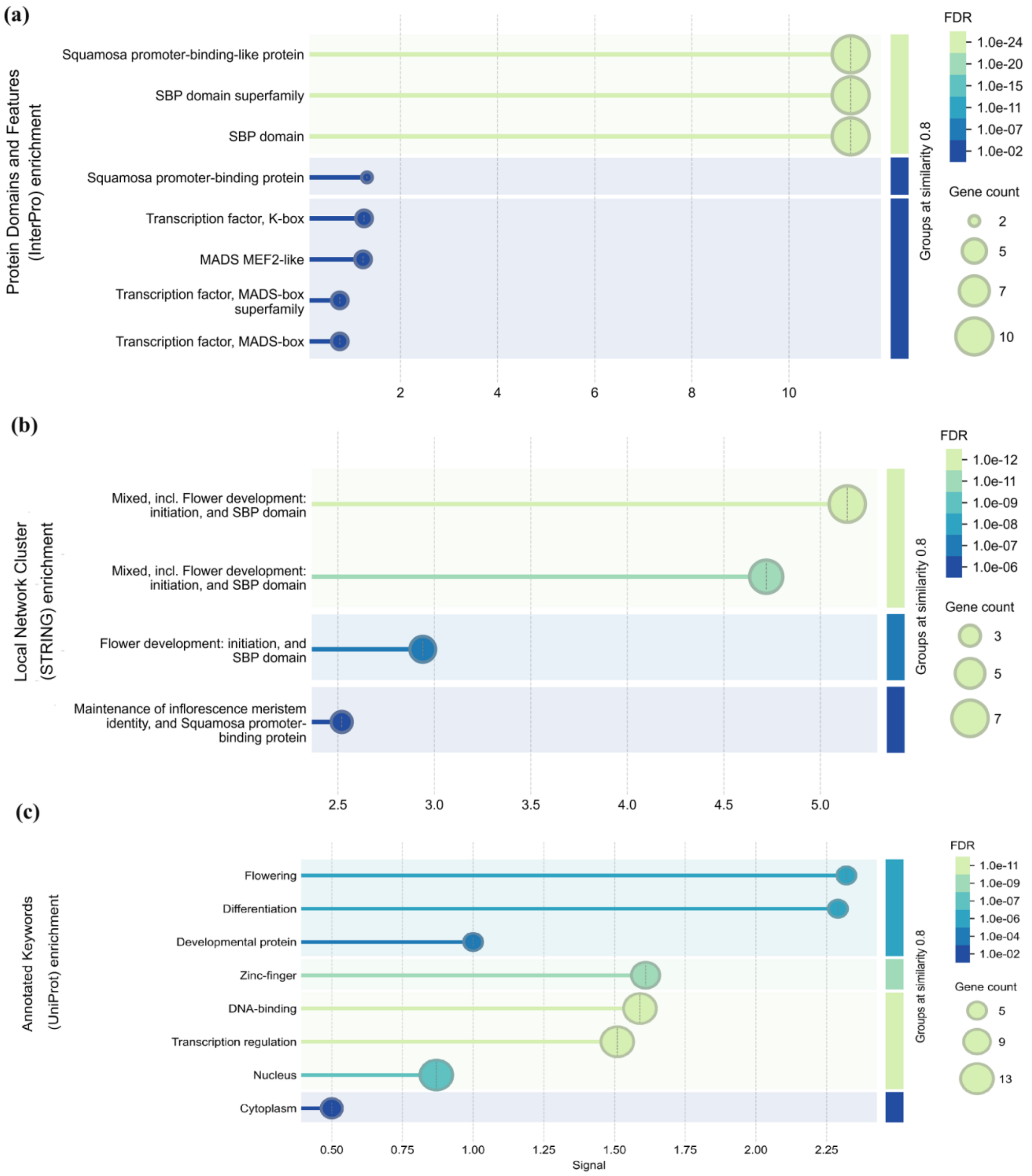

Figure S3. Protein domain, local network cluster and annotated keywords enrichment analysis of *SpSPL* gene members in spinach. The analysis was performed using three complementary approaches to identify overrepresented

biological themes. (**a**) Protein Domains and Features (InterPro) enrichment. Shows significant enrichment of DNA-binding domains characteristic of key plant transcription factor families, including the SBP (Squamosa Promoter-Binding) and MADS-box families. (**b**) Local Network Cluster (STRING) enrichment. Depicting functional clusters from protein-protein interaction networks. The size of the data points corresponds to the number of genes in the cluster, and the color represents the False Discovery Rate (FDR). Key enriched biological processes are labeled, with the most significant terms relating to flower development, initiation, and meristem identity; (**c**) Annotated Keywords (UniProt) enrichment. The chart summarizes enriched functional keywords. The dot plot shows keywords related to biological processes (e.g., Flowering, Differentiation), protein function (e.g., Developmental protein, DNA-binding), and subcellular localization (e.g., Nucleus). Dot size indicates the number of genes, and color represents the statistical significance (-log10 FDR). Collectively, these analyses robustly indicate that the gene set is highly enriched for transcription factors, particularly from the SBP and MADS-box families, that regulate flowering and floral organ development. FDR, False Discovery Rate.

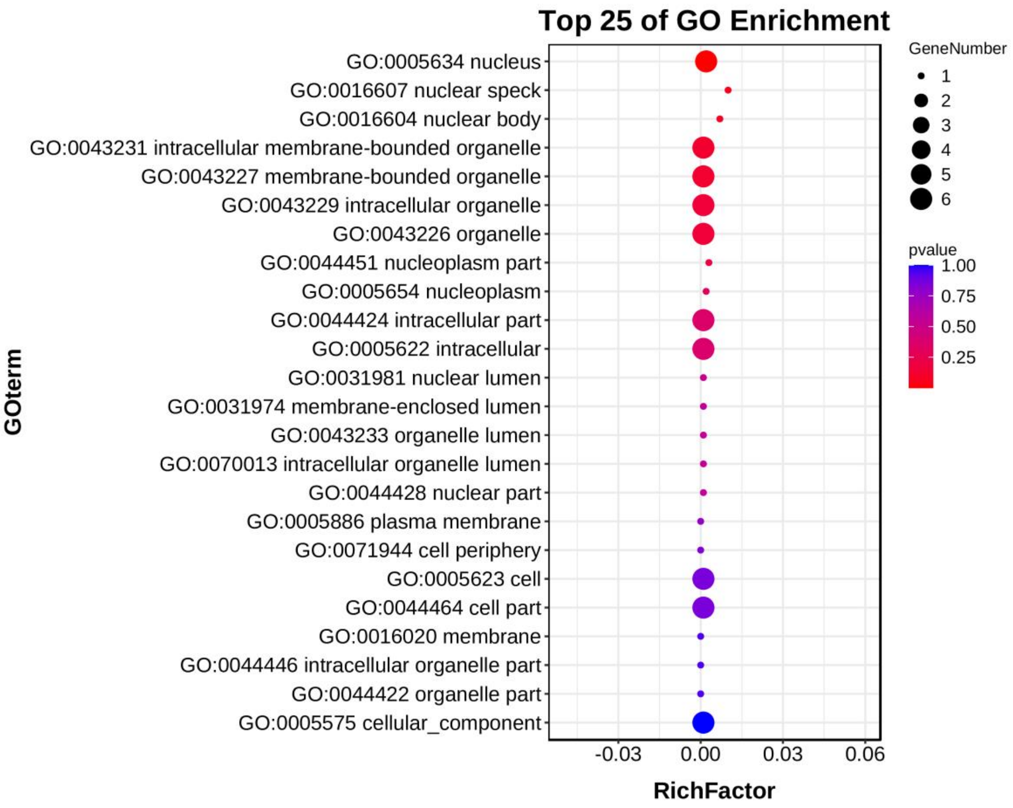

Figure S4. Top 25 GO-enrichment analyses of *SpSPL* gene members in spinach. The bubble chart displays the top 25 significantly enriched GO terms. The Rich Factor (the proportion of genes in the target set associated with the term) is plotted against the significance level -log10 (p-value). The size of each bubble corresponds to the number of genes annotated with the specific GO term (GeneNumber), and the color gradient represents the p-value, with darker shades indicating greater statistical significance. The analysis shows a strong and significant enrichment for terms related to the nucleus (e.g., nucleoplasm, nuclear speck, nuclear body) and other intracellular membrane-bounded organelles, indicating that the encoded proteins are predominantly localized to these cellular compartments.
